# Testing the Functional Profiles of School Refusal Behavior and Clarifying Their Relationship With School Anxiety

**DOI:** 10.3389/fpubh.2020.598915

**Published:** 2020-12-03

**Authors:** Carolina Gonzálvez, Ángela Díaz-Herrero, Ricardo Sanmartín, María Vicent, Aitana Fernández-Sogorb, José M. García-Fernández

**Affiliations:** ^1^Department of Development Psychology and Teaching, University of Alicante, San Vicente del Raspeig, Spain; ^2^Department of Development Psychology and Education University of Murcia, Murcia, Spain

**Keywords:** school attendance problems, school refusal, school anxiety, latent profile analysis, adolescence

## Abstract

Students with school attendance problems are a diverse and heterogeneous group whose patterns of symptomatology can change over time. This study aims to identify different school refusal behavior profiles and to determine whether these profiles differ from each other based on four situational factors and three response systems of school anxiety across gender. The participants were 1,685 Spanish students (49% female) aged 15–18 years (*M* = 16.28; *SD* =0.97). The School Refusal Assessment Scale-Revised (SRAS-R) and the School Anxiety Inventory (SAI) were administered. Latent profile analysis revealed five school refusal behavior profiles: Non-School Refusal Behavior, Mixed School Refusal Behavior, School Refusal Behavior by Positive Reinforcement, Low School Refusal Behavior, and High School Refusal Behavior. The results indicated that High School Refusal Behavior and Mixed School Refusal Behavior groups were the most maladaptive profiles since it obtained the highest mean scores on school anxiety. In contrast, Non-School Refusal and School Refusal Behavior by Positive Reinforcement groups revealed the lowest scores in school anxiety. Non-significant gender-based differences were found, only girls were more represented in the mixed school refusal behavior profile in comparison with boys but with a small effect size. Findings are discussed in relation to the importance of promoting good mental health to prevent school attendance problems in adolescents and younger ages.

## Introduction

School context plays a fundamental role in the cognitive and psychosocial development of young people. School non-attendance has been linked to numerous deficits, such as cognitive-academic, social and behavioral. Specifically, School Attendance Problems (SAPs) have been linked to poor academic performance, low scores on reading and mathematics tests, grade repetitions and even school dropout (1–4). Likewise, internalizing and externalizing behavior problems are frequent in adolescents with difficulties attending school, among which we can include anxiety, depression, substance use and pre-criminal behavior (5–10).

SAPs represent an extremely complex phenomenon, which various authors have tried to conceptualize and classify (11–13). Classification has normally distinguished between two main types of approaches: categorical and dimensional. Categorical approaches have tried to differentiate between different types of SAPs, advocating homogeneity within a category and qualitative differences between categories (14). On the other hand, dimensional approaches defend a *continuum* within SAPs and highlight heterogeneity within a category and quantitative differences between categories (14). However, Heyne et al. (11) and Kearney et al. (12, 13) affirm that these classificatory perspectives are compatible and complementary, with different but equally useful purposes that contribute to the understanding of the differences between young people with SAPs.

Part of the complexity associated to this problem lies in the causal heterogeneity of each case. Taking this premise into account and from a dimensional approach, Kearney and Silverman (15) developed the functional analysis model of school refusal behavior. This model includes four possible reasons or motives—called functional conditions—for the maintenance of SAPs in young people: (1) Avoidance of stimuli that provoke negative affectivity, (2) Escape from aversive social and/or evaluative situations, (3) Pursuit of attention from significant others, and (4) Pursuit of tangible reinforcement outside of school. In the first two functional conditions, the school refusal behavior is maintained by negative reinforcement (for example, escaping from school situations that cause discomfort or avoiding oral or written tests) and in the last two by positive reinforcement (for example, getting parents' attention or spending school time on more enjoyable activities like watching TV or playing video games). These four functional conditions can be assessed by the School Refusal Assessment Scale (SRAS) (16) and its revised version (SRAS-R) (17). Both scales have been widely used in numerous European, Asian, and American countries, revealing adequate psychometric properties [e.g., (18–26)].

School refusal behavior can be caused by various reasons at the same time but, in each particular situation, they have a different relative strength (27). This implies that it is essential to detect groups or profiles that characterize students with school refusal behaviors in order to develop prevention and intervention strategies adapted to their needs.

From this perspective, several studies have tried to establish profiles or categories of students with school refusal behavior using cluster analysis and latent class analysis (7, 8, 28–38). One of the pioneering studies that attempted to identify profiles of school refusal behaviors, based on the functional model of Kearney and Silverman (16), was carried out by Dube and Orpinas (31). These authors distinguished three groups in a non-clinical sample of 99 American students with SAPs (*M* = 12.5; *SD* = 1.38; range = 8–15 years). Specifically, the results of the cluster analysis revealed a mixed school refusal behavior group, which included explanatory factors for both positive and negative reinforcement, a school refusal behavior group for positive reinforcement, and a group of non-school refusal. Subsequent studies have sought to clarify these groups in Spanish and Ecuadorian students identifying, among the different profiles, five regular groups. Three of them coincide with Dube and Orpinas findings. However, two news groups were identified. On the one hand, the school refusal behavior profile by negative reinforcement, which is characterized by a combination of high scores in the first two functional conditions (30, 33–35). On the other hand, another consistent profile involving a group with high scores in all four functional conditions (8, 36).

In most of these investigations, the profiles detected were related to psychoeducational variables that could be associated with school refusal behavior. The empirical evidence from these studies highlighted that children and youth belonging to the School refusal behavior by multiple or mixed reinforcements, High school refusal behavior and School refusal behavior by negative reinforcement profiles were those which showed the greatest psychological and social problems, since they showed higher mean scores in anxiety, depression and stress and lower scores in social functioning and self-concept, as well as a higher risk of being able to commit or suffer cyberbullying situations (7, 8, 30, 33–36).

School anxiety is defined as a set of cognitive, psychophysiological and motor responses that people emit in school situations that are evaluated as threatening, dangerous and/or ambiguous (39). Among the school situations that can cause high levels of anxiety in young people, those related to academic and social evaluation, situations of school failure and punishment, situations involving interactions with other people, and situations of aggression and/or victimization stand out [e.g., (39, 40)]. The early identification of this problem, relatively frequent in adolescents, is fundamental due to the negative repercussions on academic, psychological and social adjustment [e.g., (41, 42)].

Numerous studies have found that anxiety disorders show comorbidity with SAPs [e.g., (24, 43–47)]. School refusers characterized by high scores in any of the first three factors of the SRAS-R or in combination have been associated with different maladaptive behaviors such as generalized anxiety disorders, social anxiety and separation anxiety (7, 20, 21, 33, 44). In contrast, school refusers by obtaining tangible reinforcements outside of school have shown not significant relationships with anxiety problems (21, 44). Similar results have been found regarding the relationship between school anxiety and school refusal behavior in Spanish children aged 8–12 years old (48–50). Students with high levels of school anxiety scored significantly higher in the first three factors of the SRAS-R. In addition, these three factors were positive and statistically significant predictor variables for high anxiety about school failure and punishment. In contrast, no significant differences were found between high and low school anxiety groups regarding the fourth factor of the SRAS-R. Differences across gender revealed that girls reported higher levels in school anxiety in comparison with boys regardless of their diagnosis (40).

More recently, Gonzálvez et al. (34) in a study carried out with 1,113 Spanish children (*M* = 9.53; *SD* = 1.10) between 8 and 12 years of age, identified profiles of school refusal behavior and examined its relationships with school anxiety. The assessment instruments used were the SRAS-R and the School Anxiety Inventory (SAI) for Children (51). The cluster analysis established four profiles: Non-school refusers, School refusers by positive reinforcement, School refusers by negative reinforcement and School refusers by mixed reinforcement. Likewise, the results of the analyses of variance highlighted that children belonging to the School refusers by mixed reinforcement category were those who showed higher mean scores in three dimensions of school anxiety (Anxiety before failure and school punishment, Anxiety before social evaluation, and Anxiety before school evaluation) and in the three anxiety response systems (cognitive, behavioral, and psychophysiological), except in the situational dimension anxiety before aggression in which the School refusers by negative reinforcement group obtained higher mean scores.

Literature review has revealed that little previous research has analyzed the relationship between school refusal behavior and school anxiety (48, 50). Thus, further research is needed in adolescent populations, considering the multidimensional nature of school anxiety construct and using more rigorous statistical methodology, such as latent profile analysis instead of cluster analysis (52). The present study sought to address these limitations with two main aims. The first aim is to verify whether there are different school refusal behavior profiles across gender with respect to the four functional conditions established by Kearney and Silverman (15) in Spanish adolescents. The second aim was to examine differences between the identified school refusal behavior profiles and their scores on school anxiety situational dimensions (Anxiety before failure and school punishment, Anxiety before aggression, Anxiety before social evaluation, and Anxiety before school evaluation) and the three anxiety response systems (Cognitive, Behavioral, and Psychophysiological) across gender. Based on prior empirical evidence, it is expected that: (1) latent profile analysis would generate four school refusal behavior profiles (Non-school refusal, School refusal by positive reinforcement, School refusal by negative reinforcement and School refusal by mixed reinforcement) (31, 34), (2) girls will achieve higher school anxiety scores than boys (40), and (3) the mixed profile of school refusal behavior would be statistically significant associated with high scores in school anxiety (34, 48, 50).

## Materials and Methods

### Participants

This was a non-interventionist transversal study with a sample made of 1,685 Spanish adolescents (49% being females) collected by random cluster sampling. The initial sample was composed by a total of 1,751 students but, 45 (2.57%) were excluded due to coding errors during the tests, 17 (0.97%) were omitted for not having the written consent of their legal tutors to participate in the research and 4 (0.23%) were excluded because not having good level of Spanish to understand the items. The participants ages range from 15 to 18 (*M*_age_ = 16,28; *SD* = 0.97) and their distribution was as follows: 411 participants with 15 years (200 boys and 211 girls), 595 participants with 16 years (310 boys and 285 girls), 474 participants with 17 years (240 boys and 234 girls) and 205 participants with 18 years (110 boys and 95 girls). No statistical age or gender differences were found (χ^2^ = 1.79; *p* = 0.62). With regard nationality, 88.7% were Spanish and the remaining participants had been born in other countries. The socio-economic level, based on the parents' labor situation and academic education levels, was considered as middle class.

### Measures

Before filling the two questionnaires used in this study, the participants filled a brief socio-demographic questionnaire providing information as age, gender and country of birth or nationality.

The school refusal behavior was assessed using the Spanish version of the School Refusal Assessment Scale-Revised (SRAS-R) (18). The original version of the SRAS-R is a 24-item self-report measure that assesses the four functional conditions for the maintenance of school refusal behavior (SRB) (17): (1) Avoidance of school related stimuli that provoke a sense of general Negative Affectivity (ANA, e.g., “How often do you stay away from school because you will feel sad or depressed if you go?”), (2) Escape from aversive Social and/or Evaluative situations at school (ESE, e.g., “If it were easier for you to make new friends, would it be easier for you to go to school?”), (3) Pursuit of Attention from Significant others (PAS, e.g., “How much would you rather be taught by your parents at home than by your teacher at school?”), and (4) Pursuit of Tangible Reinforcement outside of the school setting (PTR, e.g., “How often do you refuse to go to school because you want to have fun outside of school?”). In this study the Spanish version is made up of 18 items maintaining the four factors. Each item was scored on a seven-point Likert scale (0 = never; 6 = always) and this scale can be administered to students who range in age from 8 to 17 years. Adequate values of internal consistency have been reported, ranging from 0.70 (Factor I) to 0.87 (Factor III). In this study the coefficients of internal consistency (Cronbach's alpha) were 0.71 (Factor I), 0.76 (Factor II), 0.76 (Factor III), and 0.64 (Factor IV).

The school anxiety was assessed using the School Anxiety Inventory (SAI) (51). The SAI is a self-report measure composed of 23 items related to school situations (Factor I. Anxiety about Academic Failure and Punishment, e.g., “If I get bad marks”; Factor II. Anxiety about Aggression, e.g., “If I am insulted or threatened”; Factor III. Anxiety about Social Evaluation, e.g., “If I ask the teacher in class”; and Factor IV. Anxiety about Academic Evaluation, e.g., “Just before the exam”) and 15 items related to three response systems of anxiety (5 items Cognitive response, e.g., “I am afraid of being wrong”; 5 items Behavioral response, e.g., “I find myself without words”; and 5 items Psychophysiological response, e.g., “My breathing is fast”). This inventory can be administered to adolescents who range in age from 12 to 18 years. The SAI has a situation-response (S-R) format in which students are asked to assess the frequency with which they experience cognitive, physiological and behavioral anxiety responses in school situations using a 5-point Likert scale (ranging from 0 = Never to 4 = Always). The reliability and validity evidence based on the internal structure of SAI was satisfactory with internal consistency values (Cronbach's alpha) ranging from 0.82 (Behavioral response anxiety) to 0.93 (Anxiety about Social Evaluation) (51). In this study the coefficients of internal consistency were 0.94 (Factor I. Anxiety about Academic Failure and Punishment), 0.94 (Factor II. Anxiety about Aggression), 0.95 (Factor III. Anxiety about Social Evaluation), and 0.91 (Factor IV. Anxiety about Academic Evaluation) for each of the four school situations; and 0.83 (Cognitive), 0.85 (Psychophysiological), and 0.83 (Behavioral) for the three anxiety response systems.

### Procedure

First, the research team contacted to 17 high school principals to explain the aims of this study and ask for their collaboration. Finally, 14 high schools agreed to participate and an informed consent was then sought from the students' parents or legal guardians. The participants filled out the questionnaires anonymously in the classrooms (average time ~30 min). The questionnaires were handed out and the instructions were read out loud. All sessions were supervised by a member of the research team who had previously received instruction in the procedures. The participants were asked to answer honestly and openly and to raise a hand if they had any doubts. The Ethics Committee of the University of Alicante (code of ethics: UA-2017-09-05) approved the study, and the standards established by the Declaration of Helsinki (1964) were followed.

### Statistical Analyses

Firstly, latent profile analysis was performed to identify the subgroups of students with SRB. To determine the most adequate class solution, a series of latent profile analyses models were applied. The Bayesian Information Criteria (BIC) and the Akaike Information Criterion (AIC) were used as goodness-of-fit measures. The model with the lowest BIC and AIC values was preferred. Other two types of model data fit indices, the Vuong-Lo-Mendell- Rubin likelihood-ratio test and the bootstrap likelihood ratio test, were also used. In both cases, a *p*-value below 0.05 indicates that the estimated k-class model is better than the (k – 1)-class model, which is therefore rejected in favor of a model with at least k classes (53). In addition, entropy was used as a criterion of the quality of class membership classification whose score closer to one was preferred. Finally, it is also important to consider the size of the classes to select the best model. Classes should include at least 1% of the sample (54). Beyond these indices, theoretical feasibility and psychological significance, together with maximize the inter-classes differences of each of the groups, should be considered in selecting the best model. Mplus version 8 was used in this study because provides these statistics (55). In addition, the Statistical Package for the Social Sciences (SPSS-24) was used to identify through proportion z-test the differences in each of the clusters according to gender. The effect size used to estimate the difference in sample proportions was calculated using index *d* Cohen (56).

Secondly, a multivariate analysis of variance (MANOVA), which is a statistical technique for comparing multivariate sample means, was used. In this study it was conducted to examine the differences in the school anxiety dimensions between the SRB profiles identified and across gender. The partial eta-squared index ($\eta_{\text{p}}^{2}$) and *post-hoc* tests (Bonferroni's method) were performed to identify between which groups there were statistically significant differences. Likewise, the effect size was calculated using index *d* to obtain the magnitude of the differences observed (56). The index *d* is interpreted as follows: values between 0.20 and 0.49 indicate a low effect size, between 0.50 and 0.79 a moderate effect size, and above 0.80 a high effect size. This data was analyzed using the statistical package SPSS version 24.

## Results

### School Refusal Behavior Profiles

Table 1 presents the fit indices of the five models examined including the BIC, the AIC, the Vuong-Lo-Mendell-Rubin likelihood-ratio test, the bootstrap likelihood ratio test, the entropy information, and the number of classes that do not achieve at least 1% of the sample. The lowest BIC and AIC scores were obtained by the model of six classes. However, the more restrictive criterion was the Vuong-Lo-Mendell-Rubin likelihood-ratio test and in this case presented a *p* > 0.05. Combining all the criteria, the fifth model was selected as the best fitting model with *p* < 0.05 for the Vuong-Lo-Mendell-Rubin likelihood-ratio test and the bootstrap likelihood ratio test, the second lowest scores in the AIC and BIC indices in comparison with the rest of models, and an entropy value higher than the value of the sixth model.

**Table 1:** Data fit of all models.

**Models**	**AIC**	**BIC**	**BIC-adjusted**	**LRT**	**LRT-adjusted**	**BLRT**	**Entropy**	**Size**
2	17546.386	17616.970	17575.671	<0.001	<0.001	<0.001	0.832	0
3	17048.160	17145.892	17088.708	<0.001	0.001	<0.001	0.814	0
4	16890.538	17015.417	16942.349	0.034	0.0362	<0.001	0.780	0
5	16725.009	16877.035	16788.083	0.009	0.0108	<0.001	0.796	0
6	16630.383	16809.558	16704.721	0.120	0.1252	<0.001	0.777	0

*LRT, Vuong-Lo-Mendell-Rubin Likelihood-Ratio Test; BLRT, Bootstrap Likelihood Ratio Test*.

**Figure 1:**
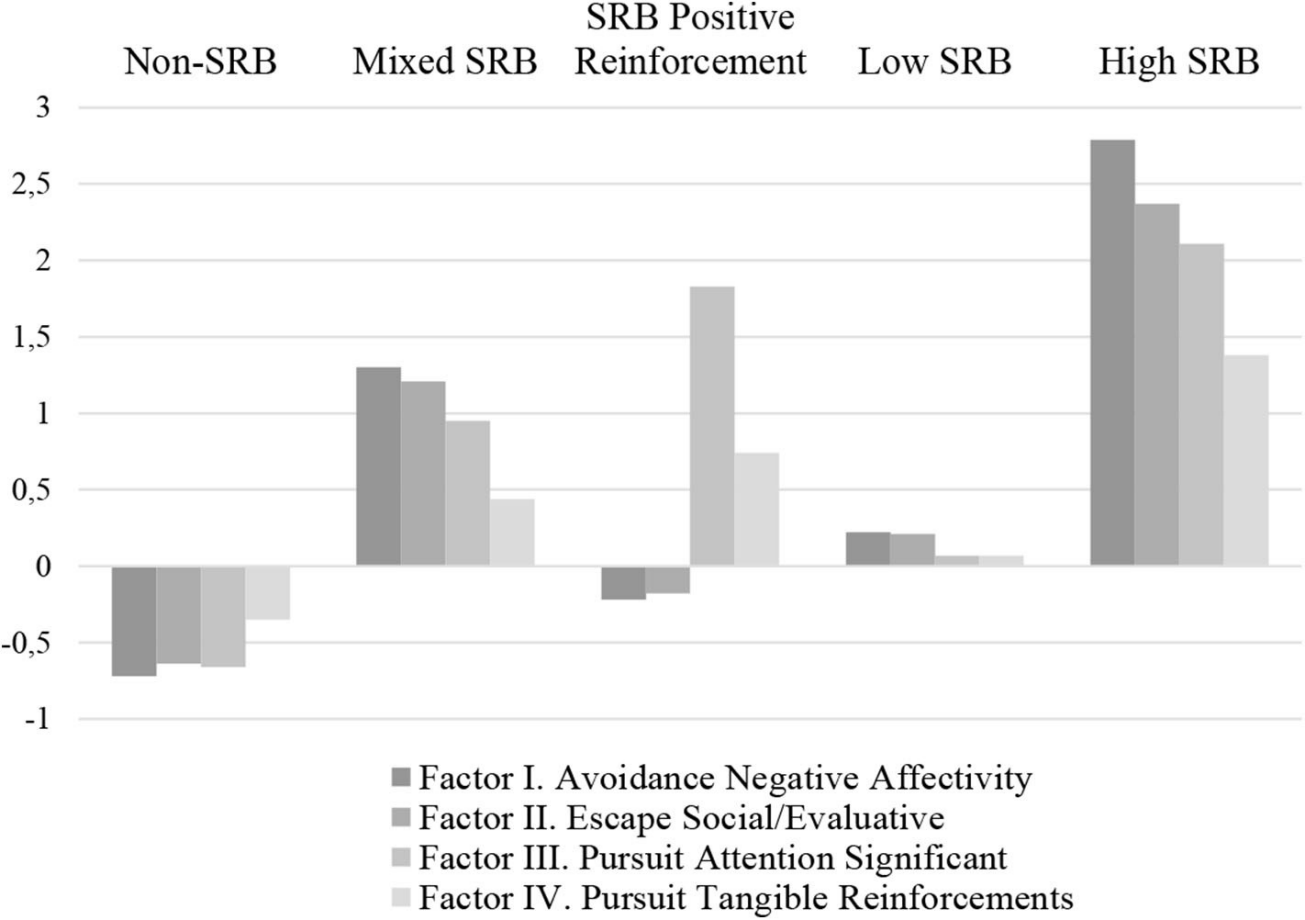
School refusal behavior profiles.

Figure 1 summarizes the five classes that were identified: Profile 1 Non-School Refusal Behavior (46.2% of the sample, with low scores in all dimensions of the SRAS-R but particularly low significant scores in the first three factors); Profile 2. Mixed School Refusal Behavior (13.1% of the sample, with high scores in the first three SRB dimensions of the SRAS-R); Profile 3. School Refusal Behavior by Positive Reinforcement (4.5% of the sample, with high scores in the last two factors of the SRAS-R); Profile 4. Low School Refusal Behavior (33.4% of the sample, with not significant scores in SRB); and Profile 5. High School Refusal Behavior (2.8% of the sample, with high SRB scores in the four dimensions of the SRAS-R).

Table 2 presents gender differences proportions in each cluster. Statistically significant differences were only found across gender in the Mixed SRB profile. In this group, girls reached a higher representation (15.3%) compared to boys (11%). However, the magnitude of the differences found was small (*d* = 0.13).

**Table 2:** Difference in each cluster proportion between boys and girls.

	**Boys**	**Girls**	***Z***	***d***
Non SRB	47.9%	44.4%	1.44	-
	(412/860)	(366/825)		
Mixed SRB	11.0%	15.3%	2.61^*^	0.13
	(95/860)	(126/825)		
SRB by	4.3%	4.6%	0.30	-
Positive Reinforcement	(37/860)	(38/825)		
Low SRB	34.4%	32.4%	0.87	-
	(296/860)	(267/825)		
High SRB	2.3%	3.4%	1.36	-
	(20/860)	(28/825)		

* *p < 0.05; SRB, School Refusal Behavior*.

### Inter-Class Differences in School Anxiety

The MANOVA results revealed statistically significant differences in the seven variables of school anxiety [Lambda de Wilks = 0.168, *F*_(28,1680)_ = 11.09; *p* < 0.001, $n_{\text{p}}^{2}$ = 0.04]. Table 3 displays means and standard deviations among the variables school anxiety and SRB profiles. Statistically significant differences were revealed between the seven SAI dimensions and the five profiles of SRB identified. The highest average scores were obtained in all cases by the High SRB group, followed by the Mixed SRB profile. On the contrary, the Non-SRB group reached the lowest average scores in the seven dimensions of the SAI.

**Table 3:** Means and standard deviations obtained by the five clusters in SAI dimensions.

**Dimensions SAI**	**Non** **SRB**		**Mixed** **SRB**		**SRB by** **positive reinforcement**		**Low** **SRB**		**High** **SRB**		**Statistical** **significance**	
	***M***	***SD***	***M***	***SD***	***M***	***SD***	***M***	***SD***	***M***	***SD***	***F_**(4, 1680)**_***	***$\eta_{p}^{2}$***
FI	39.63	29.02	68.47	39.78	44.92	35.32	58.88	34.20	81.01	40.51	57.10^*^	0.120
FII	104.44	46.88	123.85	51.31	116.38	49.51	120.87	43.57	142.39	53.95	18.09^*^	0.041
FIII	59.85	36.36	95.27	49.34	70.78	40.08	79.84	41.53	113.70	48.61	53.28^*^	0.113
FIV	38.66	20.41	57.32	27.20	45.17	26.37	48.21	21.80	65.56	25.11	45.60^*^	0.098
Cognitive	55.61	24.88	70.78	32.26	62.50	29.68	66.76	27.61	81.47	33.19	26.60^*^	0.060
Psycho.	39.04	27.24	65.85	36.61	45.02	31.71	54.42	30.54	78.66	34.75	54.96^*^	0.116
Behavioral	23.16	17.67	42.75	26.22	28.53	19.25	33.69	20.47	50.47	21.73	61.93^*^	0.129

*FI, Anxiety about Academic Failure and Punishment; FII, Anxiety about Aggression; FIII, Anxiety about Social Evaluation; FIV, Anxiety about Academic Evaluation; SRB, School Refusal Behavior; Psycho, Psychophysiological*.

* *p < 0.001*.

Considering gender distribution, the MANOVA results revealed statistically significant differences in the seven variables of school anxiety for boys [Lambda de Wilks = 0.162, *F*_(28,855)_ = 5.36; *p* < 0.001, $n_{\text{p}}^{2}$ = 0.04] and girls [Lambda de Wilks = 0.201, *F*_(28,820)_ = 6.48; *p* < 0.001, $n_{\text{p}}^{2}$ = 0.05]. Tables 4, 5 displays means and standard deviations among the variables of school anxiety and the five SRB profiles according to boys and girls, respectively. Results did not reveal gender-based differences. Both obtained the highest average scores in school anxiety by the High SRB group, followed by the Mixed SRB profile. In contrast, the Non-SRB group got the lowest average scores in the seven dimensions of school anxiety.

**Table 4:** Means and standard deviations obtained by the four clusters in SAI dimensions (boys).

**Dimensions SAI**	**Non** **SRB**		**Mixed** **SRB**		**SRB by** **positive reinforcement**		**Low** **SRB**		**High** **SRB**		**Statistical** **significance**	
	***M***	***SD***	***M***	***SD***	***M***	***SD***	***M***	***SD***	***M***	***SD***	***F_**(4, 855)**_***	***$\eta_{p}^{2}$***
FI	36.52	26.85	58.32	35.31	40.05	32.58	53.84	32.56	79.95	41.97	25.16^*^	0.105
FII	97.89	42.66	111.26	47.82	107.10	47.23	110.70	40.31	114.45	45.86	4.91^*^	0.022
FIII	53.51	33.99	85.01	46.55	62.29	42.03	71.76	39.76	101.90	50.89	22.66^*^	0.096
FIV	35.31	18.56	50.22	25.56	38.45	22.86	43.57	20.49	58.60	25.97	17.60^*^	0.076
Cognitive	53.23	23.19	63.33	27.41	58.29	28.45	62.25	26.72	72.60	35.81	8.33^*^	0.038
Psycho.	34.51	24.67	59.03	35.86	38.48	30.71	48.24	28.83	73.20	38.25	25.60^*^	0.107
Behavioral	20.23	16.44	36.24	25.94	25.00	19.21	30.88	20.12	46.95	22.79	26.51^*^	0.110

*FI, Anxiety about Academic Failure and Punishment; FII, Anxiety about Aggression; FIII, Anxiety about Social Evaluation; FIV, Anxiety about Academic Evaluation; SRB, School Refusal Behavior; Psycho, Psychophysiological*.

* *p < 0.001*.

**Table 5:** Means and standard deviations obtained by the four clusters in SAI dimensions (girls).

**Dimensions SAI**	**Non** **SRB**		**Mixed** **SRB**		**SRB by** **positive reinforcement**		**Low** **SRB**		**High** **SRB**		**Statistical** **significance**	
	***M***	***SD***	***M***	***SD***	***M***	***SD***	***M***	***SD***	***M***	***SD***	***F_**(4, 820)**_***	***$\eta_{p}^{2}$***
FI	43.13	30.95	76.13	41.35	49.65	37.62	64.46	35.15	81.75	40.19	31.20^*^	0.132
FII	111.81	50.27	133.34	51.98	125.42	50.62	132.14	44.33	162.35	50.94	13.08^*^	0.060
FIII	66.98	37.65	103.01	50.14	79.05	36.76	88.80	41.67	122.14	45.99	28.68^*^	0.123
FIV	42.44	21.72	62.67	27.26	51.71	28.17	53.36	22.08	70.53	23.69	26.08^*^	0.113
Cognitive	58.28	26.43	76.39	34.54	66.60	30.64	71.75	27.77	87.82	30.26	17.47^*^	0.079
Psycho.	44.14	29.08	70.99	36.47	51.39	31.77	61.28	30.98	82.57	32.17	27.70^*^	0.119
Behavioral	26.46	18.43	47.65	25.44	31.97	18.92	36.82	20.44	53.00	20.99	34.02^*^	0.142

*FI, Anxiety about Academic Failure and Punishment; FII, Anxiety about Aggression; FIII, Anxiety about Social Evaluation; FIV, Anxiety about Academic Evaluation; SRB, School Refusal Behavior; Psycho, Psychophysiological*.

* *p < 0.001*.

Tables 6, 7 present the *post-hoc* comparisons in general and depending on gender, respectively. The High SRB profile scored significantly higher than the Non SRB group with a high effect size in all the school anxiety dimensions assessed. These differences also occurred in the case of boys and girls, reaching a large effect size for all dimensions except, in the case of boys, for the second factor of the SAI on anxiety about aggression, whose magnitude was small. The High SRB also reported higher scores than the SRB by positive reinforcement group and the Low SRB profile whose effect size were mostly high and moderate. Similar results were found between the Mixed SRB group and the Non SRB profile, where the first one scored higher in school anxiety with an effect size mainly high and moderate in all the dimensions. On the other hand, statistically significant differences were found, but with a small and moderate effect size, between the profiles, Non SRB and Low SRB, as well as between the Mixed SRB and the SRB by Positive Reinforcement profiles. By contrast, the lowest effect sizes were found between the Mixed SRB and the Low SRB groups. No gender differences were found in these comparisons. Finally, no statistically significant differences were found between the Non SRB and the SRB by Positive Reinforcement profiles, and practically little between the Mixed and the High SRB profiles.

**Table 6:** Cohen's *d* value for *post-hoc* contrasts between cluster groups on SAI dimensions.

**Dimensions SAI**	**Profiles 1–2**	**Profiles 1–3**	**Profiles 1–4**	**Profiles 1–5**	**Profiles 2–3**	**Profiles 2–4**	**Profiles 2–5**	**Profiles 3–4**	**Profiles 3–5**	**Profiles 4–5**
FI	−0.91	-	−0.62	−1.39	0.61	0.27	-	−0.41	−0.96	−0.64
FII	−0.41	-	−0.36	−0.80	-	-	-	-	-	-
FIII	−0.89	-	−0.51	−1.45	0.52	−0.35	-	-	−0.98	−0.80
FIV	−0.84	-	−0.45	−1.29	0.45	0.39	-	-	−0.79	−0.78
Cognitive	−0.57	-	−0.43	−1.02	-	-	-	-	−0.61	−0.52
Psycho.	−0.91	-	−0.54	−1.43	0.59	0.35	-	-	−1.02	−0.78
Behavioral	−0.99	-	−0.56	−1.52	0.58	0.41	-	-	−1.08	−0.82

*Profile 1, Non School Refusal Behavior; Profile 2, Mixed School Refusal Behavior; Profile 3, School Refusal Behavior by Positive Reinforcement; Profile 4, Low SRB; Profile 5, High School Refusal Behavior; FI, Anxiety about Academic Failure and Punishment; FII, Anxiety about Aggression; FIII, Anxiety about Social Evaluation; FIV, Anxiety about Academic Evaluation; Psycho, Psychophysiological*.

**Table 7:** Cohen’s *d* value for *post-hoc* contrasts between cluster groups on SAI dimensions boys/girls.

**Dimensions SAI**	**Profiles 1–2**	**Profiles 1–3**	**Profiles 1–4**	**Profiles 1–5**	**Profiles 2–3**	**Profiles 2–4**	**Profiles 2–5**	**Profiles 3–4**	**Profiles 3–5**	**Profiles 4–5**
FI	−0.76/−0.97	-/-	−0.60/−0.68	−1.57/−1.22	0.53/0.65	–/0.31	−0.59/–	–/−0.40	−1.11/−0.83	−0.79/−0.49
FII	−0.31/−44	-/-	−0.31/−0.41	−0.39/−1.01	-/-	-/-	–/−0.56	-/-	–/−0.73	–/−0.67
FIII	−0.90/−0.87	-/-	−0.50/−0.58	−1.39/−1.44	0.50/0.32	0.30/0.32	-/-	-/-	−0.87/−1.05	−0.74/−0.69
FIV	−0.74/−0.87	-/-	−0.43/−0.50	−1.23/−1.28	0.47/0.39	–/0.39	-/-	-/-	−0.84/−0.71	−0.72/−0.77
Cognitive	−0.42/−0.63	-/-	−0.36/−0.51	−0.81/−1.11	-/-	-/-	-/-	-/-	−0.46/−0.70	−0.40/−0.57
Psycho.	−0.90/−0.86	-/-	−0.52/−0.59	−1.52/−1.31	0.60/0.55	0.35/0.30	-/-	-/-	−1.04/−0.98	−0.85/−0.79
Behavioral	−0.86/−1.04	-/-	−0.60/−0.30	−1.59/−1.43	0.46/0.49	–/49	-/-	-/-	−1.07/−1.06	−0.79/−0.79

*Profile 1, Non School Refusal Behavior; Profile 2, Mixed School Refusal Behavior; Profile 3, School Refusal Behavior by Positive Reinforcement; Profile 4, Low SRB; Profile 5, High School Refusal Behavior; FI, Anxiety about Academic Failure and Punishment; FII, Anxiety about Aggression; FIII, Anxiety about Social Evaluation; FIV, Anxiety about Academic Evaluation; Psycho, Psychophysiological*.

## Discussion

The first aim of this study was to identify the school refusal behaviors profiles from a functional approach considering gender-based differences; and, the second aim was, to analyze their relationships with school anxiety. One of its main contributions is to analyze across gender, for the first time in adolescents, the relationship between school refusal behavior profiles identified from latent analysis and school anxiety understood as a multidimensional construct.

Five profiles of school refusal behaviors were distinguished: Non-School Refusal Behavior, Mixed School Refusal Behavior, School Refusal Behavior by Positive Reinforcement, Low School Refusal Behavior and High School Refusal Behavior. The first three profiles coincided with those established in the first hypothesis. These groups showed similarity with those obtained in previous studies (31, 33, 34), although in some of these works (33, 34), along with the previous profiles, another category was also identified that referred to school refusal behavior due to negative reinforcement.

The other two profiles identified named Low School Refusal Behavior, characterized by scores that do not reach significance in any of the factors of the SRAS-R, and the High School Refusal Behavior profile, with high scores on the four factors of the SRAS-R, they were in discrepancy with those established in the first hypothesis. However, while the Low School Refusal Behavior profile appeared for the first time and it can be considered a group very similar to the Non-School Refusal Behavior profile, the group of High School Refusal Behavior had already been identified in Gonzálvez's study et al. (8). These two profiles seem to suggest that the four functional conditions of SRAS-R are not mutually exclusive and that there are relationships between them, as evidenced in the mixed or multiple profile obtained in most studies (7, 31, 33–36). Therefore, the first hypothesis could be partially confirmed, prompting the need for more research in this line.

Differences based on gender only identified that there was a higher proportion of girls in the Mixed School Refusal Behavior profile compared to boys, although the effect size was small. Despite this fact, the relationship between the school refusal behavior profiles and school anxiety has not revealed large differences according to gender, indicating that this problem affects boys and girls in similar conditions. These results partially support the second hypothesis since more girls were identified in the Mixed profile, characterized by school attendance problems due to school anxiety situations (33–36), but non-significant differences were found in the relationship between school refusal behavior profiles and school anxiety across gender.

Still on the subject of the relationship between school refusal behavior profiles and school anxiety, the data obtained is in line with the third hypothesis of the study. The Mixed School Refusal Behavior profile and the High School Refusal Behavior profile showed higher scores in all dimensions of the SAI. However, the profile that was the most maladaptive was the High School Refusal Behavior since the adolescents belonging to this group achieved the highest scores in all the situational factors and response systems of school anxiety. In contrast, the lowest scores for school anxiety (situational factors and response systems) were obtained by the Non-School Refusal Behavior group, followed by the Low School Refusal Behavior group. These results were supported by the analysis of effect sizes. In fact, when comparing the High School Refusal Behavior profile with the Non-School Refusal Behavior profile, the effect sizes were of high magnitude in all the dimensions of the SAI. Similarly, comparisons between the Mixed School Refusal Behavior and Non-School Refusal Behavior groups revealed effect sizes between moderate and high.

Likewise, the students belonging to the High School Refusal Behavior profile showed significantly higher scores in two situational factors (Anxiety before school failure and punishment, and Anxiety before social evaluation) and in the three anxiety response systems than adolescents belonging to the School Refusal Behavior by Positive Reinforcement and Low School Refusal Behavior groups with high and moderate effect sizes. Similar results were obtained when comparing the Mixed School Refusal Behavior profile and the School Refusal Behavior by Positive Reinforcement profile. In this case, adolescents belonging to the first profile revealed higher scores in school anxiety with moderate effect sizes in the majority of dimensions. The rest of the comparisons between groups either did not show significant results or were of low magnitude in some dimension of the SAI.

Consequently, as a result of these findings, adolescents belonging to the High School Refusal Behavior profile followed by the students of the Mixed School Refusal Behavior profile were those who showed higher scores in school anxiety. The adolescents belonging to these two profiles represented ~16% of the total, results consistent with the characteristics of a non-clinical sample.

Thus, these data tend to support the existence of school anxiety problems in students who base their school refusal behavior, especially on the first three factors of the SRAS-R, associated with the High School Refusal Behavior and the Mixed School Refusal Behavior profiles. These findings are in line with those obtained in other studies with children that also related the first three factors of the functional model with higher levels of school anxiety (33, 48, 50). Children who develop school refusal behavior in these groups are characterized by internalizing problems. However, other externalizing problems should be considered as temperament of truants in order to analyze its impact on the rest of the profiles. Another central point is the fact of finding that not all situational factors of school anxiety are equally associated with school refusal behaviors. In fact, anxiety about aggression was one of the factors that had the least clinical significance in the comparisons of the most maladaptive school refusal behavior profiles (High School Refusal Behavior and Mixed School Refusal Behavior) with the Non-School Refusal Behavior profile. These data suggest that students who belong to the High School Refusal Behavior and Mixed School Refusal Behavior profiles showed greater fear in school situations such as being academically evaluated through written or oral tests, having to expose in class, suffering academic failure, and be penalized by parents or teachers for misbehavior. Therefore, reducing anxiety levels and improving coping strategies in these circumstances could help to prevent, or at least decrease, the occurrence of SAPs.

This research presents some limitations that should be solved in future work. First, the results obtained cannot be generalized to other age groups or to other countries. In this sense, it would be important to corroborate these findings in other age ranges and nationalities. In addition, this study should be based on a larger and diverse sample, considering such us key variables the country of birth, differences between schools and health and psychological sample's records. Second, the cross-sectional design used in the study makes impossible to establish casual relationships. Therefore, it would be advisable to carry out longitudinal studies that provide information on the evolution of school refusal behaviors and school anxiety over the years. Third, it would be essential, in terms of methodology, for future research to adopt a multi-method and multi-source perspective. For this, other types of evaluation instruments such as diagnostic interviews, observation scales, checklists and attendance records should be incorporated, along with self-report measures, where information is collected from youth, parents and teachers. It would be interesting to complement these findings with a categorical approach based on the distinction between types of SAPs. The use of comprehensive assessment process that covers forms, types and functions of SAPs will likely provide substantial information that can be directly linked to highly individualized and effective treatment plan (11, 57). Finally, expand research to immediate environments and broader contexts which have an impact on the individual behavior is still needed (58, 59).

Despite these limitations, the results of this study are highly relevant as they provide a comprehensive pioneering analysis of the relationships between school refusal behaviors and school anxiety in adolescents. The associations found between the different profiles of school refusal behaviors and the dimensions of school anxiety can be used by education and mental health professionals as an empirical basis to develop effective preventive and intervention actions to promote school adjustment. These actions could include educational methodologies, such as tutoring or school counseling, cooperative learning and self-evaluation, as well as interventions devoted to improving the quality of the student-teacher relationship (60). In turn, relaxation training, cognitive-behavioral techniques and self-control, self-reinforcement and problem-solving techniques would be recommended to enhance the mental health of individuals with school attendance problems. The multidimensional nature of SAPs and the different SRB profiles identified may fit well with a multi-tiered system of supports (MTSS) (61). This approach includes a three-tiered system of respective supports for various absenteeism severity levels (12, 13). However, for the success of these interventions it would be essential to adopt an interdisciplinary approach including teachers, administrators, guidance counselors, school nurses, and parents' involvement (27, 31, 62).

## Data Availability Statement

The raw data supporting the conclusions of this article will be made available by the authors, without undue reservation.

## Ethics Statement

The studies involving human participants were reviewed and approved by Ethics Committee of the University of Alicante UA-2017-09-05. Written informed consent to participate in this study was provided by the participants' legal guardian/next of kin.

## Author Contributions

CG, ÁD-H, and JG-F: conceptualization. RS and MV: methodology. RS and JG-F: formal analysis. MV and AF-S: investigation. CG and ÁD-H: writing—original draft preparation. AF-S: writing—review and editing. JG-F: funding acquisition. All the authors contributed equally to the research design, data analysis, revision, and approved the final manuscript.

## Conflict of Interest

The authors declare that the research was conducted in the absence of any commercial or financial relationships that could be construed as a potential conflict of interest. The handling editor declared a past co-authorship with several of the authors CG, RS, MV, and JG-F.
